# *PDE4B* Proposed as a High Myopia Susceptibility Gene in Chinese Population

**DOI:** 10.3389/fgene.2021.775797

**Published:** 2022-01-12

**Authors:** Fuxin Zhao, Wei Chen, Hui Zhou, Peter S. Reinach, Yuhan Wang, Suh-Hang H. Juo, Zhenglin Yang, Anquan Xue, Yi Shi, Chung-Ling Liang, Changqing Zeng, Jia Qu, Xiangtian Zhou

**Affiliations:** ^1^ School of Optometry and Ophthalmology and Eye Hospital, Wenzhou Medical University, Wenzhou, China; ^2^ State Key Laboratory of Optometry, Ophthalmology and Vision Science, Wenzhou, China; ^3^ Beijing Advanced Innovation Centre for Biomedical Engineering, Key Laboratory for Biomechanics and Mechanobiology of Ministry of Education, School of Engineering Medicine, School of Biological Science and Medical Engineering, Beihang University, Beijing, China; ^4^ Center for Myopia and Eye Disease, Department of Medical Research, China Medical University Hospital, Taichung, China; ^5^ The Key Laboratory for Human Disease Gene Study of Sichuan Province and Institute of Laboratory Medicine, Sichuan Provincial People’s Hospital, University of Electronic Science and Technology of China, Chengdu, China; ^6^ Center for Myopia and Eye Disease, China Medical University Hospital, Taichung, China; ^7^ Key Laboratory of Genomic and Precision Medicine, Beijing Institute of Genomics, The Chinese Academy of Sciences, Beijing, China; ^8^ Research Unit of Myopia Basic Research and Clinical Prevention and Control, Chinese Academy of Medical Sciences, Wenzhou, China

**Keywords:** phosphodiesterase 4B (PDE4B), genome-wide association (GWA), high myopia (HM), A549 cell lines, collagen, CRISPR/Cas9, human scleral fibroblasts (HSFs)

## Abstract

Myopia is the most common cause of refractive error worldwide. High myopia is a severe type of myopia, which usually accompanies pathological changes in the fundus. To identify high myopia susceptibility genes, DNA-pooling based genome-wide association analysis was used to search for a correlation between single nucleotide polymorphisms and high myopia in a Han Chinese cohort (cases vs. controls in discovery stage: 507 vs. 294; replication stage 1: 991 vs. 1,025; replication stage 2: 1,021 vs. 52,708). Three variants (rs10889602T/G, rs2193015T/C, rs9676191A/C) were identified as being significantly associated with high myopia in the discovery, and replication stage. rs10889602T/G is located at the third intron of phosphodiesterase 4B (*PDE4B*), whose functional assays were performed by comparing the effects of rs10889602T/T deletion of this risk allele on *PDE4B* and *COL1A1* gene and protein expression levels in the rs10889602T/T^del/del^, rs10889602T/T^del/wt^, and normal control A549 cell lines. The declines in the *PDE4B* and *COL1A1* gene expression levels were larger in the rs10889602T/T deleted A549 cells than in the normal control A549 cells (one-way ANOVA, *p* < 0.001). The knockdown of *PDE4B* by siRNA in human scleral fibroblasts led to downregulation of COL1A1. This correspondence between the declines in rs10889602 of the *PDE4B* gene, *PDE4B* knockdown, and COL1A1 protein expression levels suggest that *PDE4B* may be a novel high myopia susceptibility gene, which regulates myopia progression through controlling scleral collagen I expression levels. More studies are needed to determine if there is a correlation between *PDE4B* and high myopia in other larger sample sized cohorts.

## 1 Introduction

Myopia is the most common cause of refractive error, which leads to visual impairment and even blindness (Cho et al., 2016; Flitcroft et al., 2019). Its prevalence can even exceed more than 80% in young people in China and Singapore (Sun et al., 2012; Saw et al., 2016). High myopia (HM), is defined as a refractive error that is either equal to or less than -6.00 diopters (D), which is commonly accompanied by excessive axial elongation (≥26 mm). This condition can also involve other complications, including retinal detachment, macular degeneration, cataract, and glaucoma. In such cases, these conditions are also referred to as pathological myopia (Zejmo et al., 2009). It is predicted that the prevalence of HM will progressively increase to reach 9.8% worldwide by 2050, which creates a substantial burden on providing effective health care (Holden et al., 2016). Even though there are therapeutic measures that have had some success in retarding HM development, its pathogenesis is still poorly defined. This limitation hinders development of innovative procedures that improve therapeutic management of this sight compromising condition.

There is increasing evidence that myopia progression is influenced by risk factors, which include genetics and the environment. Extensive effort is being committed to clarifying the genetic basis of HM. This endeavor includes twin studies, which determine if familial segregation and linkage mapping are associated with one another (Wojciechowski et al., 2005; Jones-Jordan et al., 2010). Genetic linkage analyses have thus far confirmed the identity of some multiple loci in chromosomal regions from MYP1 to MYP26 that are associated with myopia (Tedja et al., 2019). Moreover, genome-wide association studies (GWAS) have identified several hundred myopia candidate genes in several different ethnic cohorts including nearly a half-million participants (Tedja et al., 2018; Hysi et al., 2020). Besides myopia, several GWAS were focused on identifying the specific genetic factors associated with HM (Nakanishi et al., 2009; Li et al., 2011a; Li et al., 2011b; Shi et al., 2011; Fan et al., 2012; Meng et al., 2012; Khor et al., 2013; Shi et al., 2013). The underlying genetic mechanisms of these two conditions may be different from one another since there is little overlap between myopia (Tedja et al., 2018; Tedja et al., 2019; Hysi et al., 2020) and HM susceptibility gene loci. To clarify this question, more effort is required to identify the susceptibility genes of HM.

In the present work, we performed a DNA-pooling based genome-wide association (GWA) analysis to identify the potential susceptibility genes of HM in Chinese cohorts. Three variants (rs10889602T/G, rs2193015T/C, rs9676191A/C) were identified as candidate associations with HM. Follow-up functional assays showed that the rs10889602 (located at the third intron of the *PDE4B* gene) deletion downregulates *PDE4B* and *COL1A1* expression levels in both homozygous (rs10889602T/T^del/del^) and heterozygous (rs10889602T/T^del/wt^) A549 cell line counterparts. These findings suggest that *PDE4B* (cAMP hydrolase) may be a candidate HM susceptibility gene that increases myopia progression through downregulating scleral collagen expression levels. They are also consistent with our earlier report (Zhao et al., 2021) that losses and/or declines in *PDE4B* function decreased collagen type I expression levels through increases in cAMP accumulation, which in turn promoted myopia progression in mice and guinea pigs.

## 2 Materials and Methods

### 2.1 Human Subjects

This study was conducted in accordance with the principles of the Declaration of Helsinki and was approved by the institutional review board of Wenzhou Medical University (KYK-2015-1) as well as by ethics committees of individual hospitals. Written informed consent was collected from all participants.

In the DNA pooling-based GWA study, subjects of the Han population in southern China were recruited at the Eye Hospital of Wenzhou Medical University (WMU), First Hospital of Nantong University, and Shenzhen Eye Hospital. All subjects were given complete eye examinations, including visual acuity (Topcon RM-8800, Topcon Corp., Tokyo, Japan), spherical refractive error (KOH3 Keratometer, Nikon, Tokyo, Japan), axial length (Zeiss IOL Master, Carl Zeiss Meditec, Jena, Germany), and fundus photography (Canon CR6-45NM Fundus Camera, Canon Inc., Tokyo, Japan). The HM group consisted of 507 individuals with spherical refractive error < −8.00 diopters (D) in at least one eye. HM patients with systemic or known genetic disorders, such as Stickler Syndrome, Marfan’s Syndrome, keratoconus, and spherophakia were excluded from this study. Control subjects (*n* = 294) were selected with criteria including spherical refractive errors between −0.5 D to +2.00 D, with the axial length <24 mm in both eyes, no known ocular disease or systemic connective tissue disorder, no family history of HM, or any other genetic diseases.

In the replication studies, we used two independent sample sites. Subjects of Han descent from Kaohsiung Medical University Hospital (KMUH) in Taiwan and Sichuan Provincial People’s Hospital (SPPH) in Chengdu were recruited as the validation panel. Individuals (*n* = 2,016) were enrolled in KMUH from southern Taiwan between 2003 and 2009, all HM subjects (*n* = 991) had a spherical refraction < −6.0 D in at least one eye, and subjects with spherical refraction between −0.5 D to +1.5 D in both eyes were used as the controls (*n* = 1, 025). HM subjects (*n* = 615) who had a spherical refraction < −6.0 D in at least one eye from Chengdu in Sichuan province were selected as cases of the second sample site. And 52,708 random controls were obtained from Han Chinese Genome Database (https://www.hanchinesegenomes.org/) (Gao et al., 2020).

### 2.2 DNA Pooling-Based GWA Study and Replication

We adopted a pooling strategy for the GWA study (Macgregor et al., 2008; Bosse et al., 2009). In brief, DNA samples were mixed equally for genotyping to discover association signals based on significant differences in allele frequencies between HM and control groups. Genomic DNA was extracted from peripheral white blood cells using the QIAamp DNA Blood Mini Kit (Qiagen, Hilden, Germany). Two batches of HM pools (331 and 176 subjects) and control pools (145 and 149 subjects) were constructed from each group. Each pool contained three replicates to reduce experimental bias. All pools were genotyped on Human1M-Duo arrays following the manufacturer’s protocols (Illumina Inc., San Diego, CA). After whole-genome analysis, 751 specimens (476 cases and 275 controls) with adequate amounts of DNA in the discovery panel, were further genotyped for individual verification of pooling results. Tag SNPs were individually genotyped with MassArray (Agena, San Diego, CA, United States) that were close to loci having *p*-values above the threshold of 10^−8^.

Summary statistics of rs10889602T/G from KMUH were processed from a single replication panel, which contained 991 HM cases and 1, 025 controls. They were genotyped on the ABI 7900 Real-Time PCR System (ThermoFisher Scientific, Waltham, MA, United States). The 615 HM patients from SPPH and 476 HM patients from WMU were genotyped on the Agena MassArray platform as the case group of the Chinese mainland. The allele frequency of 52,708 random controls downloaded from the Han Chinese Genome Database (Gao et al., 2020) was used as the control group of the Chinese mainland.

### 2.3 CRISPR/Cas9 Gene Editing System Constructed Mutated A549 Cell Lines Containing the rs10889602

GWAS delineated the rs10889602T/G SNP as being associated with HM. It is located on chromosome 1 in the third intron region of *PDE4B*. At this site, a CRISPR/Cas9 gene-editing tool replaced the *PDE4B* gene sequence chr1: 66, 573, 331–66, 573, 462 [∼130 base pair (bp), including rs10889602 site] based on the human genome reference (hg19) with the PuroR cassette (Beijing Biocytogen Co., Ltd., Beijing, China). Fibroblasts (especially human derived) would have been the optimal option for validating the role of rs10889602T/G in modulating collagen expression levels, but some of them are difficult to culture before and/or after gene editing and/or with low PDE4B mRNA and protein expression levels. To mitigate this limitation, we chose instead A549 cell lines (which consistently express high PDE4B mRNA and protein expression levels) to generate viable rs10889602T/T deleted cells. Genotyping of rs10889602 in the normal A549 cell line was firstly identified by Sanger sequencing to eliminate the effect of rs10889602T/G or rs10889602G/G on function of the A549 cell line. Briefly, the genomic DNA of the A549 cell line was extracted using the QIAamp DNA Blood Mini Kit (Qiagen) according to manufacturer instructions. PCR amplification was carried out and generated a 413 bp product with the forward primer sequence: 5′-TGA​ACT​TGG​GTT​GTT​GAT​GT-3’; the reverse primer sequence: 5′-CAC​CTG​GTG​GCT​TAG​AAT​AG-3’. Then, the purified PCR product was sequenced on the Applied Biosystems platform (ABI 3730, Foster City, CA, United States). With this substitution, we generated homozygous (rs10889602T/T^del/del^) and heterozygous (rs10889602T/T^del/wt^) cell lines. Normal control A549 cells and rs10889602T/T deleted A549 cells were cultured in Dulbecco’s modified Eagle medium (DMEM, ThermoFisher Scientific) supplemented with 10% (v/v) fetal bovine serum (FBS, ThermoFisher Scientific), 1% GlutaMAX™ (ThermoFisher Scientific), and maintained at 5% CO_2_, 95% air humidified incubator at 37°C. The A549 cells were seeded and allowed to adhere to tissue culture plates for 24 h before treatment. Quantitative real-time polymerase chain reaction (qRT-PCR) and Western blot analysis determined their PDE4B mRNA and protein expression levels, respectively.

### 2.4 siRNA-Mediated Knockdown of *PDE4B* in HSFs

To analyze the contribution of PDE4B on scleral remodeling, the effect was determined of its downregulation on collagen protein expression levels in human scleral fibroblasts (HSFs). In the same way, genotyping of rs10889602 for HSFs was also identified by Sanger sequencing according to the description of the A549 cell line. Subsequently, we compared the effects of culturing on this response under control conditions with those in the *PDE4B* siRNA transfected counterpart. HSFs were transfected with siRNA (10 μM) by using Lipofectamine RNAiMAX (ThermoFisher Scientific) according to the typical RNAiMAX transfection procedure. Two human *PDE4B* siRNA oligos [siRNAs were designed and obtained from GenePharma (Shanghai) Co., Ltd., Shanghai, China]. They were siRNA1: 5′-UGG​AAG​ACC​UGA​ACA​AAU​GTT-3′, siRNA2: 5′-GUU​CUU​CUC​CUA​GAC​AAC​UTT-3’. In all experiments, a validated scrambled sequence (5′-AAC​GGC​CAC​AAG​UUC​AGC​GUG-3′) was used as negative control (NC), only with the supplied transfection reagents which served as the mock condition. Western blot analysis of the *PDE4B* protein expression levels was performed to determine the knockdown efficiency of siRNA, and Western blot analyses also evaluated the effects of *PDE4B* knockdown on collagen type I (COL1A1) protein expression levels.

### 2.5 Quantitative Real-Time Polymerase Chain Reaction

The A549 cells were seeded in 24-well tissue culture plates, then total RNA was extracted with 500 μl TRIzol reagent (ThermoFisher Scientific) according to the manufacturer’s instructions. Then, the total RNA (800 ng) was reverse transcribed into cDNA with Random Primers and Moloney Murine Leukemia Virus (M-MLV) Reverse Transcriptase and RNAs in Plus RNase Inhibitor (#N2615, Promega, Madison, WI, United States) after being treated with RQ1 RNase-Free DNase (#M6101, Promega). Quantitative real-time PCR was performed with the specific primers and Power SYBR Green PCR Master Mix (#4367659, ThermoFisher Scientific) on an ABI 7500 Real-Time PCR system. Each sample was run in duplicate in a final volume of 15 µl containing 1 µl cDNA, 0.5 µl (10 µM) of each primer (purchased from Shanghai HuaGen Biotech Co., Ltd., Shanghai, China), 7.5 µl of 2× Power SYBR Green PCR Master Mix and 6 µl RNase-free water. Cycling parameters of the qRT-PCR were as follows: 50°C for 2 min, 95°C for 10 min, followed by 40 cycles at 95°C for 15 s, 60°C for 60 s, and 1 cycle at 95°C for 10 min, 65°C for 60 s. After the reaction, the expression level of each target mRNA relative to that of glyceraldehyde-3-phosphate dehydrogenase (*GAPDH*) in A549 cells were obtained by the 2^−ΔΔCT^ method. These primer sequences referred to above are provided in Supplementary Table S1.

### 2.6 Western Blotting

The A549 cells and HSFs were seeded in 12-well tissue culture plates and were lysed in the radio-immunoprecipitation assay buffer (RIPA, #P0013B, Beyotime Biotechnology, Shanghai, China) supplemented with Complete Mini (protease inhibitor cocktail) and 1 mmol/l phenylmethylsulfonyl fluoride (PMSF, # ST506, Beyotime Biotechnology). Proteins were quantified using an Enhanced bicinchoninic acid (BCA) Protein Assay Kit (#P0010, Beyotime Biotechnology). An equal amount of protein samples (20 μg) was subjected to 8% sodium dodecyl sulfate-polyacrylamide gel electrophoresis (SDS-PAGE) and subsequently transferred onto a nitrocellulose membrane (#HATF00010, Millipore, Billerica, MA, United States). After blocking with 5% nonfat milk for 2 h at room temperature, the membranes were incubated with the indicated primary antibodies overnight at 4°C. The membranes were washed three times with TBST (tris-buffered saline with 0.5–1% Tween-20), then probed with dilution 1:2000 IRDye 800CW goat anti-rabbit IgG (#926-32211, Odyssey, Lincoln, NE) or IRDye 800CW goat anti-mouse IgG (#926-32210, Odyssey) or dilution 1:2,000 goat anti-rabbit IgG conjugated to HRP antibodies for 2 h at room temperature. The membranes were again washed three times with TBST as before, followed by visual analysis using Odyssey software or the electrochemiluminescence software. Finally, the protein bands were quantitated by densitometric analysis with ImageJ software (NIH, Bethesda, MD, United States). Loading equivalence was confirmed by using *β*-actin as an internal control for at least three independent experiments. Primary antibodies were used against PDE4B (1:1,000; #SAB2107176, Sigma, Milwaukee, WI, United States), and *β*-actin (1:1,000; #A5441, Sigma).

### 2.7 Animal Care and Ethics and Form Deprivation Myopia Mice Model

The C57BL/6J mouse (*Mus musculus*) came from the Animal Breeding Unit at Wenzhou Medical University. Animal studies were approved by the Animal Care and Ethics Committee at Wenzhou Medical University (Wenzhou, Zhejiang, China). The treatment and care of animals were conducted in accord with the Association for Research in Vision and Ophthalmology Statement for the Use of Animals in Ophthalmic and Visual Research. All experimental animals were raised in standard mouse cages with a 12-h light/dark cycle (light on at 8:00 a.m., light off at 8:00 p.m.), chow and water were available ad libitum.

The C57BL/6J mice (male, 21-day-old) were subjected to monocular form deprivation by covering their right eyes with a diffuser goggle glued at the periphery of the face (Huang et al., 2018). Before and after 4-weeks of form deprivation treatment, refraction and ocular biometrics of mice were measured. An eccentric infrared photoretinoscope was used to measure ocular refraction in unanesthetized mice in a dark room (Huang et al., 2018), Ocular biometric measurements, including corneal thickness, anterior chamber depth, lens thickness, vitreous chamber depth, corneal radius of curvature, and axial length, were measured by real-time optical coherence tomography (OCT) using a custom-built OCT instrument (Huang et al., 2018).

### 2.8 Immunofluorescence

Following 4 weeks of form deprivation treatment, all C57BL/6J mice were sacrificed with CO2. Eyes were enucleated and cut into an eyecup followed by fixation in 4% paraformaldehyde in 0.1 mol/L phosphate-buffered saline (PBS), pH 7.4, for 30 min, they were then immersed in 10% followed by 20% sucrose for 2 hours, respectively, and then immersed in 30% sucrose for 24 h. Eyecups were embedded in frozen section medium Neg50 (ThermoFisher Scientific), followed by rapid freezing in liquid nitrogen. They were then sectioned (10 μm) vertically on a freezing cryostat. The sections were blocked with 10% normal goat serum in 0.3% Triton X-100/PBS for 30 min at room temperature, and then incubated with rabbit polyclonal antibodies against PDE4B (#SAB2107176; Sigma, Milwaukee, WI, United States) for 24 h at 4°C. After rinsing with PBS, sections were incubated with a cyanine dye 3-labeled secondary antibody (1:2000, Jackson ImmunoResearch, West Grove, PA, United States) for 1 h at room temperature. Nuclei were stained using 4′, 6-diamidino-2-phenylindole (DAPI, 50 µg/ml, Jackson ImmunoResearch) for 5 min, follow-up mounting with phenylenediamine. A ZEISS 710 (Carl Zeiss Microimaging GmbH, Jena, Germany) microscope was used to view the sections.

### 2.9 Statistical Analysis

#### 2.9.1 Quality Control of Pooling-Based Genotyping

The allelic frequency of each replicate was approximated based on the signal intensity of each bead on the chip as described in previous studies (Macgregor et al., 2008; Bosse et al., 2009). Briefly, probes labeled with cyanine dye 3 (Cy3) and cyanine dye 5 (Cy5), respectively, hybridized the two alleles of each designed SNP. The Illumina iScan (Illumina Inc., San Diego, CA, United States) collected the fluorescent intensities and they were designated as Xraw and Yraw, respectively. After normalization of Cy3 and Cy5 intensities, the allele frequency of each SNP was calculated with the relationship f_alleleA = Xraw/(Xraw + Yraw). Simultaneously, Xraw + Yraw was used to calculate the total SNP signal intensity.

By comparing the estimated allelic frequencies, this procedure calculated the correlation coefficient (R) between pooled replicates. Using 0.99 as the cutoff, arrays with *R* < 0.99 were discarded. After performing quality control, data from one chip (1 HM and 1 control pool) were excluded, and the estimated allelic frequency of each pool was obtained by averaging the allelic frequencies of all the remaining replicates. Loci with low hybridization signal intensities may induce errors in estimates of allelic frequency. For obtaining quality control estimates, we ranked all the loci based on their total Xraw + Yraw, and then removed SNPs within the lowest 5% percentile. SNPs with diverse allelic frequencies greater than 5% between replicates, as well as loci with minor allelic frequency (MAF) lower than 1% in control pools, were also precluded from further analysis as previously described (Macgregor et al., 2008; Bosse et al., 2009). Finally, allelic frequencies of 812, 150 SNPs were utilized for association analysis.

#### 2.9.2 Association Analysis

Association analysis was conducted using a previously described formula for Z_comb_ (Macgregor et al., 2008; Bosse et al., 2009). By quantile-quantile plotting (QQ plot), Z_comb_ was identified to conform with a normal distribution pattern with a standard deviation equal to 0.771. The final *p*-value of each SNP was produced by combining standard normal distribution transformed Z_comb_ from two batches of case and control pairs. Population stratification analysis was performed with the genomic control test from randomly selected 20, 000 SNPs (Devlin and Roeder, 1999). The plot of the association result was generated by LocusZoom (Boughton et al., 2021).

For 9 individual genotyped SNPs in the verification stage, loci with call rates >0.8 were selected for association analysis using PLINK 1.07 (Purcell et al., 2007), and age and gender were set as the covariates. In the replication step, allele counts of the 9 tested SNPs in the case group were obtained by combining genotyping results of the WMU and SPPH cohort. Allele counts in the control group were calculated from allele frequencies and the total sample size of each SNP from the Han Chinese Genome Database (Gao et al., 2020). Association results were obtained by chi-square test of allele counts in case and control groups. Odds ratios and confidence intervals were also calculated accordingly. Haplotype analysis was performed using Haploview 4.1 (Barrett et al., 2005). The meta-analysis of the summary statistics of the two replication sample groups was performed by PLINK 1.07 using the inverse-variance-weighted effect-size method (Purcell et al., 2007).

#### 2.9.3 Statistical Analysis

All data are presented as mean ± standard error (mean ± SEM). Statistical Product and Service Solutions (SPSS) 20.0 software (IBM, Armonk, NY, United States) was used for statistical analysis. Multiple comparisons of *PDE4B* mutated and normal control A549 cell lines were done using one-way ANOVA with Bonferroni post hoc test. Comparisons were done of the effects of *PDE4B* siRNA transfection group and NC group in HSFs using one-way ANOVA with Bonferroni post hoc test. *p*-value < 0.05 was considered statistically significant (*p < 0.05, **p < 0.01 and ***p < 0.001).

## 3 Results

### 3.1 Pooling-Based Genome-wide Association Identifies *PDE4B*, With Genome-Level Significance, as a Potential Predisposition Gene of HM in a Chinese Cohort

The discovery and replication phases of GWA determined if there is a putative association between SNPs and HM. This was done in a case-control study involving a Han Chinese population composed of different cohorts. In the discovery phase, susceptibility genes were identified by performing genome-wide screening of pooled DNA samples from 507 HM cases and 294 controls. After verifying individual candidate SNPs, a replication study determined the most predominant 9 SNPs in two separate Han Chinese cohorts.

To minimize sample heterogeneity in the discovery phase of the GWA study, HM subjects were only included if their refractive errors were ≤ −8.00 D. The clinical features of all the enrolled HM and control individuals are summarized in Table 1. Most of these HM patients presented with degenerative changes specific to pathological myopia in the ocular fundus. The Illumina Human 1 M-Duo platform used nearly 1.2 M SNP probes to genotype genomic DNA that was obtained from HM cases and controls (see Material and Methods).

**TABLE 1:** Sample summary in discovery panel and replications.

High myopia group										Control group		
Analysis	Sample size	Age	Age of onset	Male/Female	Refraction (Diopter)		Axial length (mm)		Familial/Sporadic	Sample size	Age	Male/Female
					Right eyes	Left eyes	Right eyes	Left eyes				
GWA study	507	36.7 ± 0.7	14.7 ± 0.5	190/317	−14.95 ± 0.27	−14.62 ± 0.30	29.76 ± 0.12	29.55 ± 0.14	91/416	294	22.15 ± 0.3	166/128
Verification	476	36.8 ± 0.7	14.3 ± 0.5	177/299	−15.05 ± 0.28	−14.57 ± 0.31	29.83 ± 0.12	29.55 ± 0.14	77/399	275	22.26 ± 0.3	152/123
KMUH	991	21.21 ± 0.12	10.79 ± 0.08	659/306	−7.65 ± 0.05	−7.39 ± 0.05	—	—	331/435	1,025	21.38 ± 0.19	874/146
SPPH	615	36.03 ± 0.6	—	249/353	−9.71 ± 0.23	−9.50 ± 0.22	26.87 ± 0.15	26.71 ± 0.13	—	—	—	—

Data are Mean ± SEM; mm, millimeter.

**FIGURE 1:**
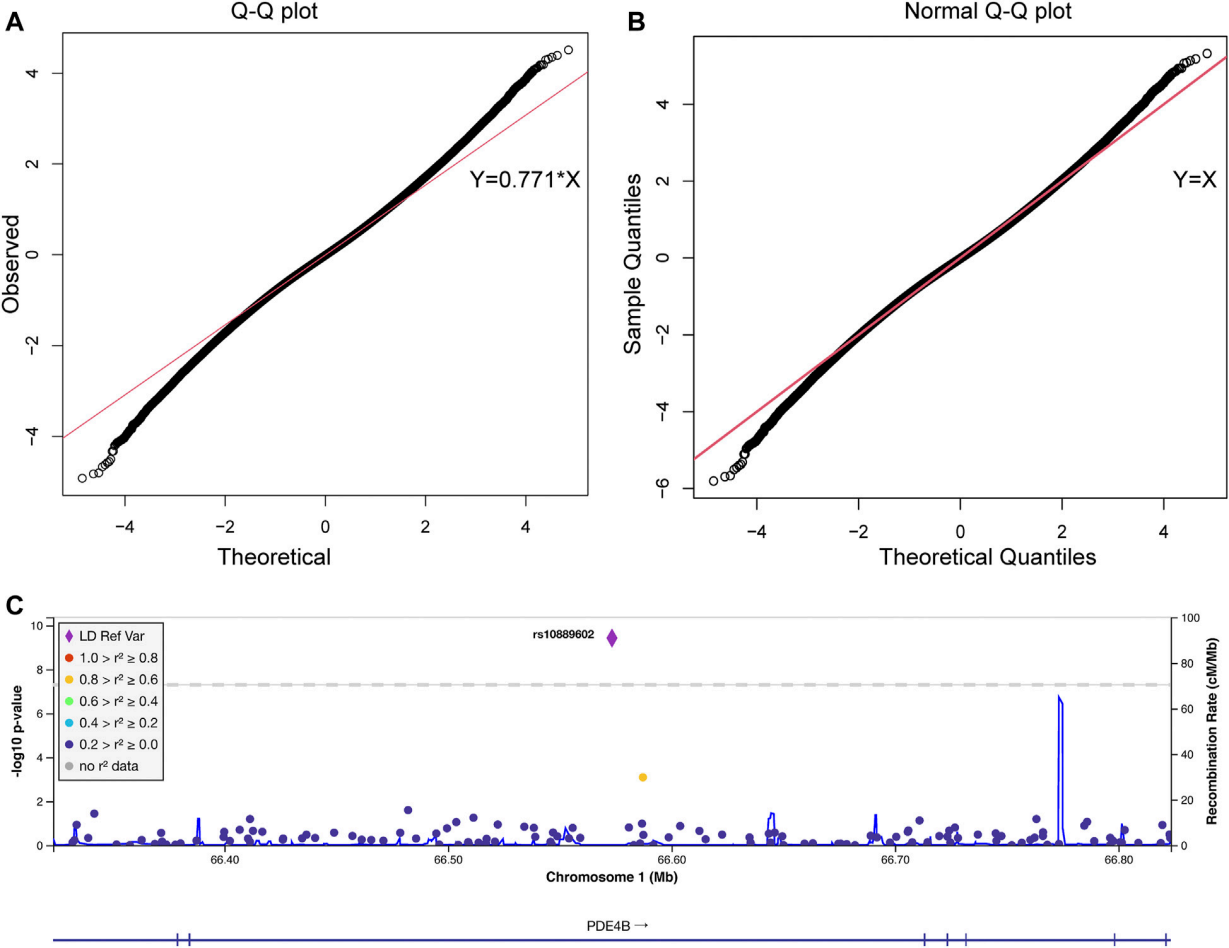
The quantile-quantile plot of the pooling-based genome-wide association analysis and the association results of the *PDE4B* region. The quantile-quantile (QQ) plots were drawn before **(A)** and after **(B)** standard normal distribution transformation. The *X*-axis indicates the observed quantiles of the Z score in our analysis, and the *Y*-axis indicates the quantiles of the Z score under a standard normal distribution. The red line indicates the standard line passes through the first and third quartiles. The formula of the standard line in **(A)** was Y = 0.771*X, which indicates our results conform with a normal distribution with a standard deviation equal to 0.771. **(C)** Association signals in the *PDE4B* region. The plot was drawn by LocusZoom. The *X*-axis indicates the physical position of each genotyped SNP according to the human genome reference (hg19). The left *Y*-axis indicates the log transformed *p*-value, which was used for labeling the dots and rhombus, and the right *Y*-axis indicates the recombination rate, which was used for labeling the blue lines in the main figure. The rhombus indicates rs10889602T/G. The color of the dots represents the linkage disequilibrium with rs10889602T/G measured by *R* ^2^ (legend at left).

As shown in the QQ plot, Z_comb_, the observed significance level, conformed to a normal distribution pattern with a standard deviation equal to 0.771 (Figure 1). The genomic inflation factor was calculated as 1.1, which indicates a population stratification in our samples. The final *p*-value of each SNP locus was obtained by transforming it to a standard normal distribution and then correcting with the genomic control score. Nine SNPs had significantly different allelic frequencies between the HM and control groups (*p* < 10^−8^, Table 2). To provide additional support of our pooling approach, we compared the estimated allele frequency of the 9 SNPs in the pooled controls in the WMU cohort with the allele frequency data from the Han Chinese Genome database (Gao et al., 2020). As shown in Supplementary Table S2, the pooling methods tend to overestimate the allele frequency. In 4 loci, there was a significant difference between the estimated and queried allele frequencies. Accordingly, to verify the pooling-based genotyping results, these nine SNPs were then individually genotyped by Agena Mass Array using the samples that had adequate DNA amounts after pooling (476 cases of HM and 275 controls) in the WMU cohort. Except for one SNP genotyping failure, there was a correlation between all of the remaining eight loci and HM occurrence (*p* < 0.05, Table 2). As shown in Table 1, as GWAS pooling revealed significant gender stratification, we also evaluated the effect of gender stratification on the association results in the above indicated individual genotyping verification step in the WMU cohort. As shown in Table 2, we found that gender stratification had little effect on the association results.

**TABLE 2:** SNPs showing association signals in GWA study and replications.

Chr	Pos^a^	SNP ID	Gene^b^	Risk allele	Discovery	Verification					Validation^d^			
					Pooling *P*	MAF-case	MAF-control	*P*_Tends_	*P*_adj_ ^c^	Or (95%CI)	MAF-case	MAF-control	P	Or (95%CI)
1	53,532,606	rs11580093	*PODN*	C	3.24 × 10^−9^	4.21%	1.80%	0.0136	0.0214	2.32 (1.13, 4.75)	3.39%	3.21%	0.662	1.06 (0.85, 1.31)
1	66,573,381	rs10889602	*PDE4B*	G	3.59 × 10^−10^	7.35%	4.30%	0.0198	0.0201	1.78 (1.10, 2.91)	6.92%	5.46%	0.0035	1.28 (1.08, 1.52)
4	73,676,690	rs1346132	*COX18*	G	1.60 × 10^−10^	5.80%	2.20%	1.60×10^−3^	2.37×10^−3^	2.70 (1.42, 5.13)	NA	NA	NA	NA
6	16,877,190	rs7762018	*PHF10*	A	4.59 × 10^−9^	32.77%	24.18%	3.27×10^−4^	5.76×10^−4^	1.53 (1.21, 1.94)	32.2%	29.9%	0.0125	1.11 (1.02, 1.21)
11	86,402,877	rs600242	*ME3*	G	4.84 × 10^−9^	17.12%	10.30%	4.10×10^−4^	5.28×10^−4^	1.78 (1.29, 2.48)	18.4%	17.6%	0.476	1.04 (0.93, 1.16)
12	21,047,074	rs4149152	*SLC O 1B3*	G	4.36 × 10^−10^	10.64%	5.30%	3.54×10^−4^	5.86×10^−4^	2.09 (1.36, 3.22)	8.75%	8.14%	0.280	1.08 (0.94, 1.24)
12	97,563,086	rs2193015	*NEDD1*	C	1.87 × 10^−9^	37.29%	48.10%	4.81×10^−5^	5.072×10^−5^	1.57 (1.26, 1.94)	40.4%	43.2%	0.0051	1.12 (1.03, 1.21)
17	76,429,546	rs618324	*DNAH17*	C	1.30 × 10^−9^	NA	NA	NA	NA	NA	NA	NA	NA	NA
18	49,625,503	rs9676191	*DCC*	C	2.68 × 10^−9^	17.87%	10.03%	9.35 × 10^−5^	1.12 × 10^−4^	1.91 (1.41, 2.58)	16.0%	13.2%	4.36 × 10-^5^	1.25 (1.12, 1.39)
	Replications of rs10889602										4.00%	5.24%	0.021	1.32 (0.98, 1.78)
	KMUH										Pmeta = 5.98 × 10^−4^	1.29		

a Based on human reference genome build 37 (NCBI, 37).

b Showing the nearest RefSeq Genes.

c *p*-value adjusted by gender and age in individual samples in the WMU, cohort.

d Combined cases from WMU, and SPPH, 50K Han genome from Han Chinese Genome Database as controls. MAF, minor allele frequency; OR, odds ratio; CI, confidence interval; NA, not available.

We further performed validation of the eight SNP in 1,091 HM cases (615 from SPPH and 476 from WMU, Table 1) and 52,708 random controls from the Han Chinese Genome Database (Gao et al., 2020). As shown in Table 2, three SNPs (rs10889602T/G, rs2193015T/C, rs9676191A/C) showed significant association with HM (*p* < 0.0056, Bonferroni correction with 9 tested SNPs). Notably, among these loci, rs10889602T/G is localized at the third intron of the *PDE4B* gene which modulates cAMP (Figure 1C). We previously showed that mediated cAMP degradation and sustained collagen type I synthesis (Tao et al., 2013; Zhao et al., 2021). Accordingly, the association signal of rs10889602 was further replicated in independent Han Chinese samples made up of 991 HM cases and 1,025 controls from KMUH. We detected a statistically significant correlation between rs10889602T/G and HM [*p* = 0.021, Odds Ratio (OR) = 1.32, 95% Confidence Interval (CI) = 0.98–1.78, Trends-test, Table 2]. Furthermore, the meta-analysis showed that there is a significant association between rs10889602T/G and HM (*P*_*meta*_ = 5.98×10^−4^, Table 2), suggesting that *PDE4B* may be a HM susceptibility gene. The detection power in such sample size was estimated as ∼0.99 by Genetic Power Calculator (http://zzz.bwh.harvard.edu/gpc/cc2.html). Altogether, the results of both the discovery and replication studies showed that *PDE4B* may be a potential HM susceptibility gene.

**FIGURE 2:**
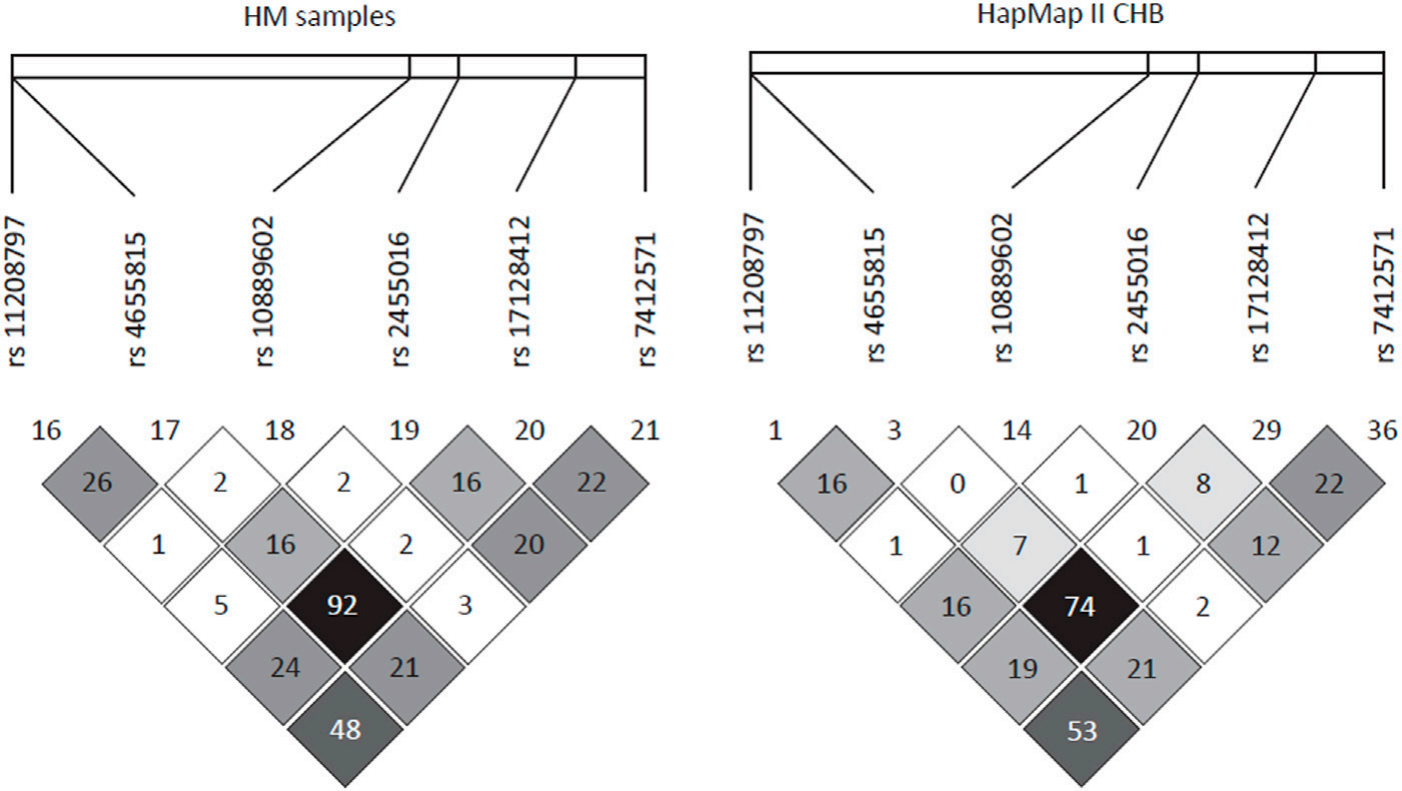
Linkage disequilibrium pattern of the region neighboring rs10889602T/G. In a 30-kb region encompassing rs110889602T/G, there was a very low linkage disequilibrium between this locus and surrounding SNPs in our samples (*r* ^2^ < 0.03, left panel) and in Han Chinese in Beijing (CHB) samples of the HapMap project (*r* ^2^ < 0.02, right panel).

To perform fine mapping of the HM susceptibility loci in *PDE4B*, we also analyzed 13 SNPs including 6 tag SNPs flanking rs10889602T/G by 30 kb and 7 SNPs in the known functional domains of *PDE4B* in the WMU cohort. However, these SNPs were unrelated to HM (*p* > 0.05) over this span. Furthermore, haplotype analysis also demonstrated that indeed there was no linkage disequilibrium (LD) between rs10889602T/G and any other surrounding SNPs in our samples (*r* ^2^ < 0.03, Figure 2). Similarly, this SNP also showed low LD with nearby SNPs in HapMap data of the Han Chinese cohort described in Beijing (Figure 2).

**FIGURE 3:**
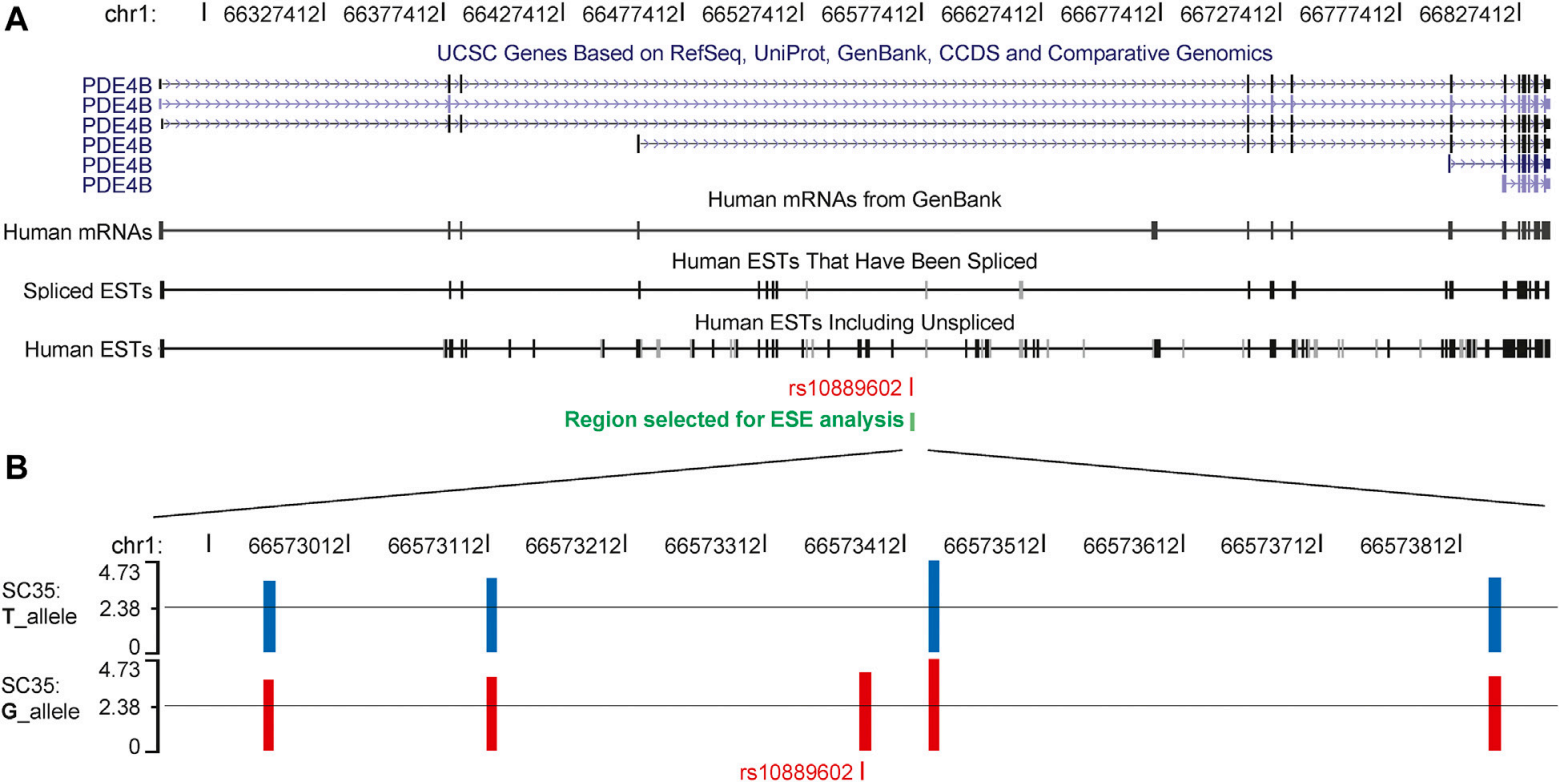
Splicing products of *PDE4B,* the G allele of rs10889602T/G generates a new SC35 binding site in the third intron. **(A)** Six isoforms and various types of expressed sequence tags of *PDE4B* have been reported in the database of the University of California at Santa Cruz Genome Browser. **(B)** In a 1,000 bp region encompassing rs10889602T/G, ESEfinder predicted either 4 or 5 binding sites for the splicing factor, SC35, with likelihood scores greater than 3.500 for each chromosome. For the chromosome bearing the T allele, from left to right, the scores of the SC35 binding sites were 3.64, 3.80, 4.73, and 3.82, respectively. An extra binding site with the likelihood score of 4.001 was predicted in the sequence containing the risk allele guanosine **(B)**. The suggested threshold of the ESEfinder was 2.383 as shown by the black lines in each plot. ESEfinder, Exon Splicing Enhancer Finder; SC35, serine and arginine rich splicing factor 2.

Exon Splicing Enhancer Finder (ESEfinder) is a web resource used to identify exonic splicing enhancers (Cartegni et al., 2003; Smith et al., 2006), which was used to perform an in silico splicing prediction of rs10889602. The HM risk allele G of rs10889602T/G was predicted to provide an extra binding site for the serine and arginine rich splicing factor 2 (SC35, Figure 3). To deal with the possibility that different isoforms of *PDE4B* contributed to mediating different functions, we examined the *Pde4b* isoform expression pattern in various tissues of the mouse. Among four isoforms of *Pde4b*, the *Pde4b-1/2* mRNA expression level was higher than that of *Pde4b*-3 in the myocardium and the brain (Supplementary Figure S1). *Pde4b-3* mRNA expression was about a hundred times higher in the spleen and kidney than *Pde4b-1/2*. In the cornea, retina, and sclera, *Pde4b-3* mRNA expression was also higher than *Pde4b-1/2*. *Pde4b-4* mRNA expression was at a lower level in the brain, spleen, and kidney, but it was absent in the myocardium, cornea, and sclera. We speculated that rs10889602T/G SNP may modulate the *Pde4b* isoform expression pattern and ultimately the total amount of *Pde4b* expression, and in doing so give rise to different biological effects.

### 3.2 Rs10889602T/T Deletion Reduces PDE4B and COL1A1 Expression Levels in A549 Cell Lines

Firstly, the genotype of the SNP (rs10889602) in the A549 cell line and HSFs obtained by Sanger sequencing identified the SNP (rs10889602) as TT in both cell lines (Supplementary Figures S2A, S2B), which is the major genotype in the population, but neither were rs10889602T/G nor rs10889602G/G. To clarify the functional influence of rs10889602 T/G on *PDE4B*, we analyzed the expression quantitative trait loci at this SNP in the Genotype-Tissue Expression (GTEx) project (eGTEx Project, 2017). Accordingly, there is no reported expression of a quantitative trait loci connection between rs10889602T/G and *PDE4B.* This negative result may be caused by the extremely low allele frequency of this locus irrespective of the presence or absence of scleral tissue in this project. Given this possibility, it was pertinent to use an alternative approach to validate that this SNP affects PDE4B expression and its enzymatic activity. Accordingly, the CRISPR-Cas9 genome editing system was used for this purpose. *PDE4B* mutants (rs10889602T/T SNP deletion), including heterozygous (rs10889602T/T^del/wt^) and its homozygous (rs10889602T/T^del/del^) counterpart were constructed in human A549 cells (see Material and Methods). Both of these two mutated cell types were cultured along with the unmodified control A549 cells.

**FIGURE 4:**
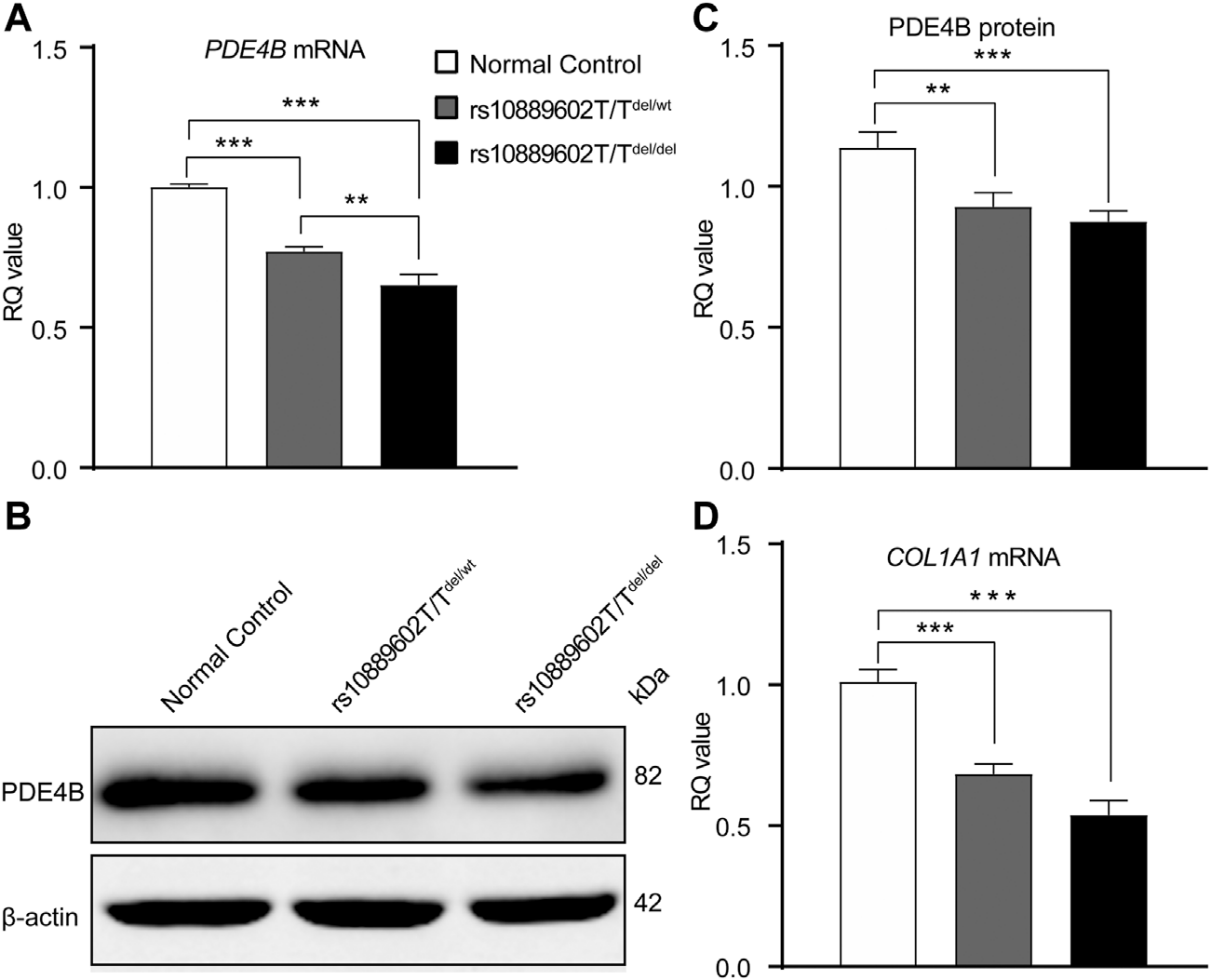
Rs10889602T/T SNP deletion in A549 cells decreases PDE4B and COL1A1 mRNA and/or protein expression levels. **(A)** qRT-PCR analysis of *PDE4B* mRNA expression levels. *GAPDH* was the loading control. **(B)** Western blot analysis of PDE4B protein expression levels, *β*-actin was the loading control. **(C)** Densitometric quantification of western blot results of PDE4B expression levels. **(D)** qRT-PCR analysis of *COL1A1* mRNA expression levels, *GAPDH* was the loading control. Data are expressed as mean ± standard error (mean ± SEM), *n* = 6 for each group, multiple comparisons of different A549 cell lines were done using the one-way ANOVA with Bonferroni post hoc test. *p*-value < 0.05 was considered statistically significant (**p < 0.01 and ***p < 0.001). qRT-PCR, quantitative real-time polymerase chain reaction; *GAPDH*, glyceraldehyde-3-phosphate dehydrogenase; RQ, relative quantity.

As is shown in Figure 4A, relative to its expression levels in A549 normal control cells, the *PDE4B* mRNA expression levels in both rs10889602T/T^del/del^ cells and in rs10889602T/T^del/wt^ cells were downregulated (rs10889602T/T^del/del^ *vs* rs10889602T/T^wt/wt^, *p* < 0.001; rs10889602T/T^del/wt^ *vs* rs10889602T/T^wt/wt^, *p* < 0.001, one-way ANOVA with Bonferroni correction). The *PDE4B* mRNA expression level in the rs10889602T/T^del/del^ cells was lower than that in rs10889602T/T^del/wt^ cells (*p* < 0.01, one-way ANOVA). Similarly, the changes in the PDE4B protein levels paralleled the declines that occurred in the mRNA level (rs10889602T/T^del/del^ *vs* rs10889602T/T^wt/wt^, *p* < 0.001; rs10889602T/T^del/wt^ *vs* rs10889602T/T^wt/wt^, *p* < 0.01, one-way ANOVA, Figures 4B,C). These significant reductions in *PDE4B* mRNA and protein expression levels in the rs10889602T/T deleted A549 cells showed that the rs10889602 variant influences the function of the *PDE4B* gene. As reported elsewhere, the downregulation of collagen along with extracellular remodeling results in scleral thinning and underlies myopia formation. Hence, we compared the effects of homozygous and heterozygous rs10889602T/T deletion on *PDE4B*-induced *COL1A1* gene expression levels in A549 cells. The mRNA levels of *COL1A1* in both rs10889602T/T^del/wt^ and rs10889602T/T^del/del^ A549 cells decreased in comparison with those in normal control A549 cells (one-way ANOVA, *p* < 0.001, Figure 4D), respectively. Therefore, the rs10889602 deletion downregulated PDE4B mRNA and protein expression, which in turn decreased the collagen type Ⅰ (*COL1A1*) mRNA expression level.

### 3.3 *PDE4B* Knockdown Suppresses COL1A1 Protein Expression in HSFs

**FIGURE 5:**
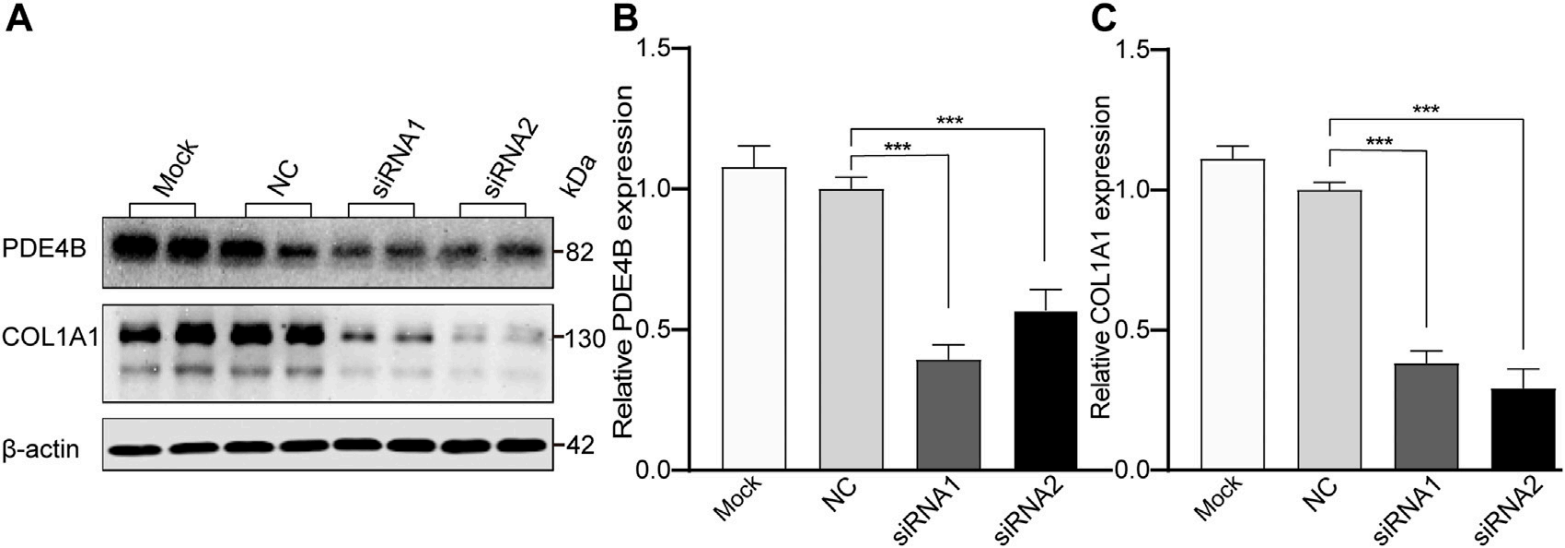
The Effect of P*DE4B* Knockdown on Expression of Type I collagen (COL1A1) in cultured HSFs. The HSFs were treated with siRNA for 48 h, then the knockdown efficiency of PDE4B and the expression of COL1A1 were analyzed by Western blot **(A)**. **(B,C)** The bar graphs represent relative protein levels of PDE4B and COL1A1. **(B)** The protein expression level of PDE4B. **(C)** The protein expression level of COL1A1. Mock: blank control (transfection reagent only); NC, negative control (cells transfected with scrambled siRNA). *β*-actin was the loading control. Data were expressed as mean ± standard error (mean ± SEM), *n* = 5 for each group. Comparison of the *PDE4B* siRNA-transfected group and scrambled siRNA group were determined by using one-way ANOVA. *p-*value < 0.05 was considered statistically significant (***p < 0.001).

To clarify the role of PDE4B in controlling scleral remodeling, which is an important modulator of myopic development, we knocked down *PDE4B* expression and analyzed its effect on the collagen type I (COL1A1) protein expression level. In HSFs transfected with *PDE4B*-siRNA for 48 h, this procedure significantly inhibited its protein expression level compared with the NC group (ANOVA, *p* < 0.001, Figures 5A,B). Accompanying these declines, COL1A1 protein expression levels also declined (ANOVA, *p* < 0.001, Figures 5A,C). Therefore, changes in PDE4B affect collagen type I expression levels in HSFs may disrupt scleral fibril fine structure and in turn induce scleral extracellular matrix remodeling.

To clarify whether the collagen was only induced in cells with the highest *PDE4B* expression level, we also analyzed the *PDE4B* mRNA background expression levels in normal control A549 cell lines (rs10889602T/T) and HSFs (rs10889602T/T). The results showed that the *PDE4B* mRNA expression level in HSFs was only 23% of its value in the normal control A549 cell line (independent sample *t*-test, *p* < 0.001, Supplementary Figure S3). *PDE4B* (relative low expression) knockdown in the HSFs also resulted in downregulation of the collagen I (COL1A1) expression level, which indicated that the PDE4B mRNA and/or protein background expression level in cells didn’t impact on its role in modulating the collagen I expression level.

### 3.4 Four Weeks of Form Deprivation Downregulates PDE4B Protein Expression Levels

After 4-weeks of form deprivation treatment, the refractive state in those eyes of the C57BL/6J mice (*n* = 6) was significantly shifted towards myopia compared with the contralateral (fellow) eyes (*n* = 6). The average dioptric shift in the form deprived eyes was -6.54 ± 0.39 D (independent samples *t*-test, *p* < 0.05, Supplementary Figure S4A). Similarly, the vitreous body depth, the axial length of form deprived eyes elongated more than that in the fellow eyes (independent samples *t*-test, *p* < 0.05, Supplementary Figures S4B, S4C). These changes confirmed establishment of form deprivation myopia (FDM) in this mouse model. To ascertain if changes in PDE4B expression levels contribute to FDM development, its immunofluorescence was evaluated in the sclera (Supplementary Figures S4D, S4E); the posterior scleral PDE4B immunofluorescence was significantly downregulated in the form deprivation-treated eyes (Supplementary Figure S4E) relative to its level in the untreated fellow eyes (Supplementary Figure S4D). This difference confirms that FDM development is associated with downregulation of scleral PDE4B protein expression levels.

## 4 Discussion

In this study, we used an unbiased pooling-based GWA approach of Chinese HM samples and controls to identify genetic associations with HM, which led to the identification of three potential HM associations, including one that lies in the intron of *PDE4B* gene. However, none of the variants passed genome-wide significance in the replication stage, possibly due to small sample sizes. These variants are needed to be further validated in larger sample sizes and/or different ethnic populations. Deletion of the rs10889602 SNP decreases PDE4B and COL1A1 mRNA and/or protein expression levels in A549 cells. Knockdown of *PDE4B* with siRNA transfection downregulated COL1A1 protein expression levels in HSFs. PDE4B protein level was downregulated in 4 weeks’ form deprivation-treated eyes in mice. These results indicate that *PDE4B* may be a HM susceptibility gene through modulating scleral collagen expression levels. Furthermore, they agree with our previous conclusion that loss or inhibition of PDE4B leads to rises in cAMP accumulation that are accompanied by declines in collagen type I expression levels and increases in myopia development (Zhao et al., 2021).

In recent years, GWA studies have been widely used for identifying myopia susceptibility loci and genes that are involved in controlling myopia progression and refractive error development. The largest GWA study contained over half a million participants and identified 438 susceptibility loci of myopia (Hysi et al., 2020). According to the most recent GWAS-catalog collection (access date: 2021-8-19), there are 1,045 susceptibility loci that were associated with refractive error or myopia (Buniello et al., 2019). Among these loci, only 80 are related to HM. Moreover, there is little overlap between HM and myopia that is shared by two SNPs or eight genes. This observation suggests that HM and myopia may be controlled by different genetic mechanisms. Even though many myopia studies had large sample sizes, they did not target HM susceptibility. In the current study, we provided support for the *PDE4B* gene being a HM susceptibility gene whose function is dependent on the expression of a designated SNP, rs10889602T/G. On the other hand, analysis of additional SNPs flanking 30 kb of this SNP and in the known functional domains of *PDE4B* in the WMU samples failed to establish any association with the control of HM gene function (*p* > 0.05). Haplotype analysis also showed no correlation between rs10889602T/G and surrounding SNPs in our samples (*r* ^2^ < 0.03) and low linkage disequilibrium with nearby SNPs in the HapMap data of Han Chinese in Beijing. This low linkage disequilibrium of rs10889602T/G with nearby SNPs may explain why we detected only one significant SNP in this gene and why previously no *PDE4B* signal was detected. One possibility is that no probe for this SNP was included in the chips utilized in earlier studies and the imputed genotype may exhibit low accuracy due to the lack of linkage disequilibrium with surrounding SNPs (Pei et al., 2008; Tedja et al., 2018; Hysi et al., 2020). According to data from the 1,000 genome project, the allele frequency of rs10889602T/G varies dramatically among different populations which is the highest in African (∼30%) and the lowest in European (∼2%). This variability indicates that the effect of this SNP on HM may differ among populations. Moreover, according to the United Kingdom Biobank (www.ukbiobank.ac.uk/), the allele frequency of rs10889602T/G is 0.01 in European descendants (Canela-Xandri et al., 2018), which is much lower than the Han Chinese value (i.e. 0.053 according to Han Chinese Genome Database) (Gao et al., 2020). These results indicate that this SNP may modulate function in a population dependent manner, which may explain why no signals were observed in the recent GWAS that contained more than half a million samples (Hysi et al., 2020). Since Africans have the highest allele frequency of the HM risk G of rs10889602, additional studies are warranted to analyze the effect of this SNP on myopia frequency in Africans.

Population stratification is an important confounding factor of association studies. Our association analysis is composed of three steps, including pooling-based whole genome association discovery step, individual genotyping of positive loci for verification step, and replication of positive loci in independent cohorts’ step. The population stratification in the discovery step was estimated by the genomic inflation factor and the result of *λ* = 1.1 indicated a stratification in these samples. Because limited SNPs were genotyped in verification and validation steps, we cannot adjust population stratification in these data. The samples in the verification step were the same as the discovery step, which indicates *λ* = 1.1 may also be suitable for adjustment. In the validation step, the cases were mainly from Zhejiang and Sichuan provinces which belong to south China, and the controls were from the whole Chinese mainland. The genetic divergence between south and north China may bring stratifications into the validation step (Xu et al., 2009). Accordingly, more HM samples from north China should be added in future work to further prove the association between *PDE4B* with HM.

Myopia has a prevalence of more than 80% in young people in China, which makes it difficult to obtain adequate non-myopic control samples in this study. In the discovery step of our study, we adopted a stringent control definition with the refractive error between −0.5D to +2.0D (Materials and Methods), which makes the control group be an extreme phenotype of the entire population and restricts the sample size to only 60% of the cases. As reported elsewhere, when dealing with extreme phenotypes, the case-control ratio of 1:0.67 has a little statistical power reduction in comparison with the ratio of 1:1 (Li et al., 2019). Hence, the smaller sample size of the control group should have limited influence on our results.

*PDE4B* variants have been reported that are mainly associated with some psychiatric disorders, such as schizophrenia (Fatemi et al., 2008; Kähler et al., 2010; Guan et al., 2012), depression (Numata et al., 2009), and panic (Malakhova et al., 2019). Some of these phenotypes also appear in *Pde4b*-KO mice (Siuciak et al., 2008; Rutten et al., 2011). As we previously reported, *Pde4b*-KO mice exhibit myopia phenotypes, which further supports our contention that it has a HM susceptibility role (Zhao et al., 2021). In addition, the fact that PDE4B inhibition induces myopia and exacerbates FDM (Tao et al., 2013; Zhao et al., 2021) together with its genetic susceptibility role provide additional indications that loss of *PDE4B* gene function serves as a noteworthy model for evaluating the interaction between genetic and environmental factors in myopia development. These findings also suggest that there are possible common pathways (such as PDE4B/cAMP signaling) underlying mild and high myopia, induced by a genetic predisposition or exposure to an environmental factor.

*In silico* splicing prediction using ESEfinder (Cartegni et al., 2003; Smith et al., 2006) indicates that the guanosine risk allele in rs10889602T/G appears to generate an extra binding site for SC35. This observation suggested that myopia formation may be the result of changes in *PDE4B* splicing, leading to an altered isoform expression/pattern in the sclera. Consistent with this notion, based on the database of the University of California at Santa Cruz (UCSC) Genome Browser (http://genome.ucsc.edu/, hg19), alternative splicing of *PDE4B* generates up to six or more isoforms. Our analysis of different *Pde4b* isoforms in the mouse sclera showed that *Ped4b-3* is highly expressed in the cornea, retina, and sclera compared with the mRNA expression levels of *Pde4b-1/2*, and *Pde4b-4,* which are absent in the cornea and the sclera. To clarify whether the rs10889602 region influences the function of *PDE4B*, we adopted the CRISPR/Cas9 genome editing system to delete this region from the A549 cells, which has the highest PDE4B protein expression level among all the available cell lines. The results showed that declines in PDE4B mRNA and protein levels accompanied decreases in collagen type in this mutated cell line. As the A549 cell line was derived from a male patient, we determined whether gender influences the association of rs10889602T/G with HM. By setting gender as a covariate in the verification stage, we find gender stratification has little effect on the association results (Table 2). Although we cannot identify the gender of each sample in the two replication groups, the results from the verification stage suggest the effects of the rs10889602 on both genders.

As reported elsewhere, PDE4B upregulation can increase collagen synthesis, which leads to lung fibrosis (Selige et al., 2011) and liver fibrosis (Cai et al., 2018). Taken together, it is evident that rs10889602T/G influences *PDE4B* function in the sclera of humans. This finding prompted us to assume that downregulation of PDE4B by deletion of this HM risk allele may lead to the declines in scleral collagen secretion, which renders the sclera more extensible and matches the excessive increases in optical axis elongation that result in myopia development. The major limitation of our work is lacking rs10889602T/G artificial point mutated cells to directly measure the relationship among changes in the expression of the HM risk allele, *PDE4B* isoforms, and total expression level. Future studies employing these constructs are needed to test the validity of this abovementioned assumption that there is a direct relationship between changes in PDE4B function, scleral collagen type I (COL1A1) protein expression levels and myopia progression.

In summary, *PDE4B,* a cAMP hydrolase is a putative HM susceptibility gene based on the results of both human GWA analyses combined with the effects of *PDE4B* deletion on collagen I (COL1A1) mRNA and protein expression levels in the *PDE4B* mutated A549 cell lines and *PDE4B* knockdown by siRNA lead to downregulation of COL1A1 protein in HSFs. Our findings demonstrate that the rs10889602T/G risk allele suppressed *PDE4B* function, which may be a genetic susceptibility factor underlying HM development in the Chinese cohorts enrolled in this study. Taken together with our previous report that loss and inhibition of PDE4B can induce myopia in mice and guinea pigs (Zhao et al., 2021), *PDE4B* may be a novel HM susceptibility gene, which may serve as a target for improving therapeutic management of myopia.

## Data Availability

The raw data supporting the conclusion of this article will be made available by the authors, without undue reservation.
