# Supplementary material for: PDE4B Proposed as a High Myopia Susceptibility Gene in Chinese Population
Source: Front Genet. 2022 Jan 12;12:775797. doi: 10.3389/fgene.2021.775797 (PMC8804583; doi:10.3389/fgene.2021.775797)
Supplement: Supplementary file 1 [file DataSheet1.docx]

**Title:** *PDE4B* Proposed as a High Myopia Susceptibility Gene in Chinese Population

**Supplementary materials**

**Figures and Tables:**


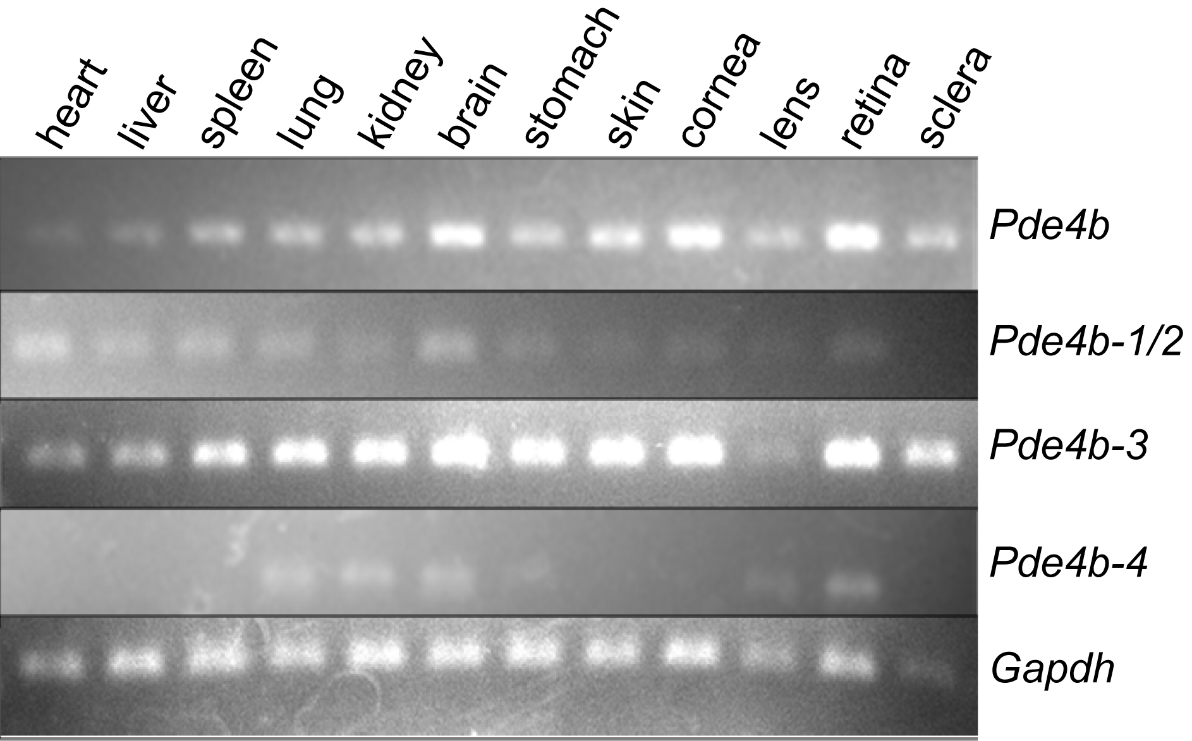


| Mouse tissue | *Pde4b-1/2* | *Pde4b-3* | *Pde4b-4* |
| --- | --- | --- | --- |
| heart | 1 | 0.322939 | 0 |
| liver | 1 | 73.13832 | 0.027877 |
| spleen | 1 | 225.1381 | 0 |
| lung | 1 | 90.32165 | 0.05726 |
| kidney | 1 | 125.5687 | 0.420448 |
| brain | 1 | 0.252088 | 0.008759 |
| stomach | 1 | 5.256127 | 0.040808 |
| skin | 1 | 68.49848 | 0 |
| cornea | 1 | 18.3622 | 0 |
| lens | 1 | 0 | 0 |
| retina | 1 | 1.173106 | 0.113807 |
| sclera | 1 | 4.993322 | 0 |

**FIGURE S1 | Expression patterns of *Pde4* isoform in mouse tissues.** *Pde4b-1/2* mRNA expression was higher than *Pde4b-3* in the myocardium and brain. *Pde4b-3* mRNA was highly expressed in the spleen and kidney at levels that were about a hundred times higher than *Pde4b-1/2*. In the cornea, retina, and sclera, *Pde4b-3* mRNA was also expressed higher than *Pde4b-1/2*. *Pde4b-4* mRNA was expressed at lower levels in the brain, spleen, and kidney, and it was absent in the myocardium, cornea, and sclera. *Gapdh* as an internal control.


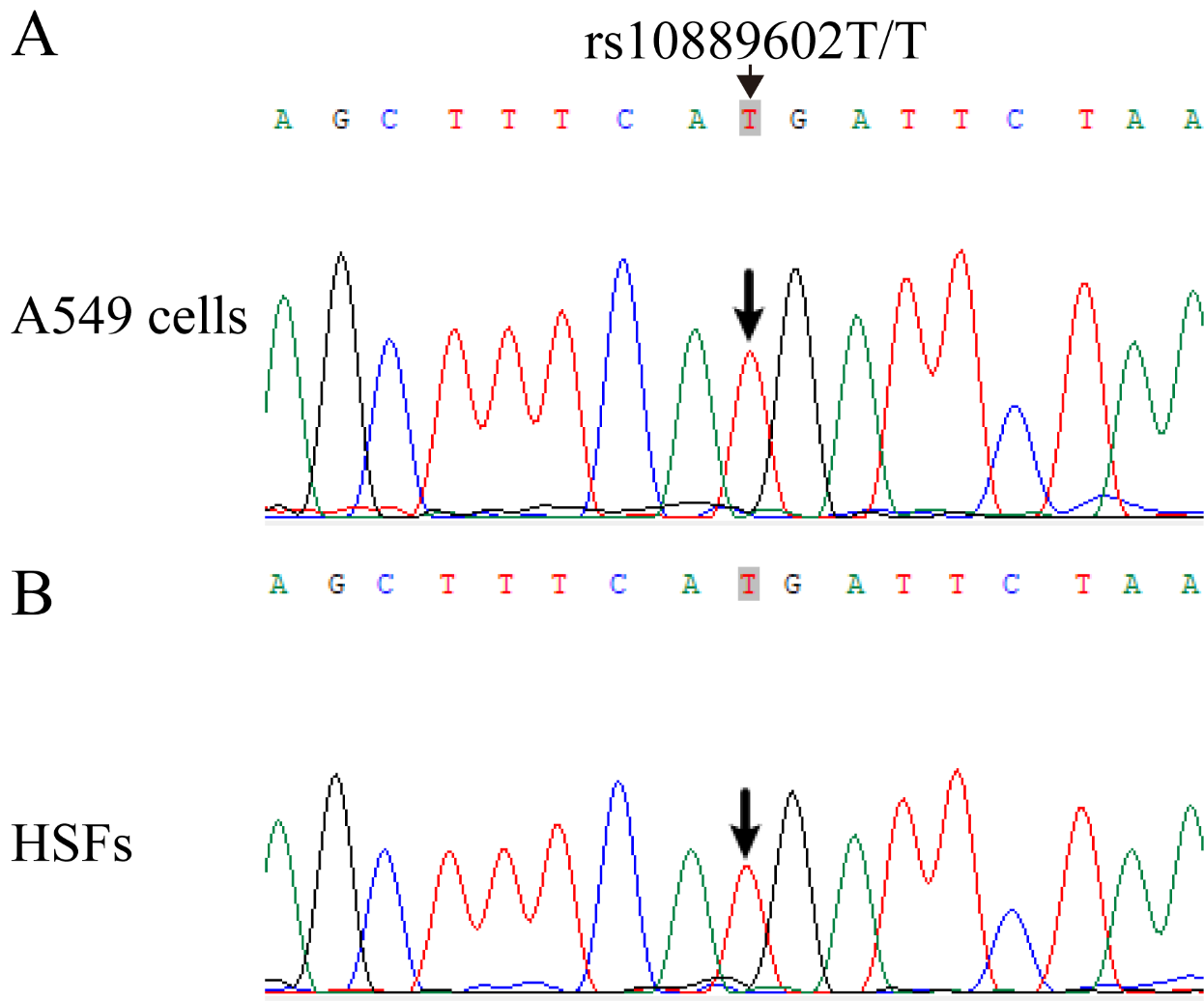


**FIGURE S2** **|** **The chromatogram of rs10889602 was obtained by Sanger sequencing in A549 cells and HSFs.** The chromatogram showed that the SNP (rs10889602) is TT in A549 cells (**A**) and HSFs (**B**).


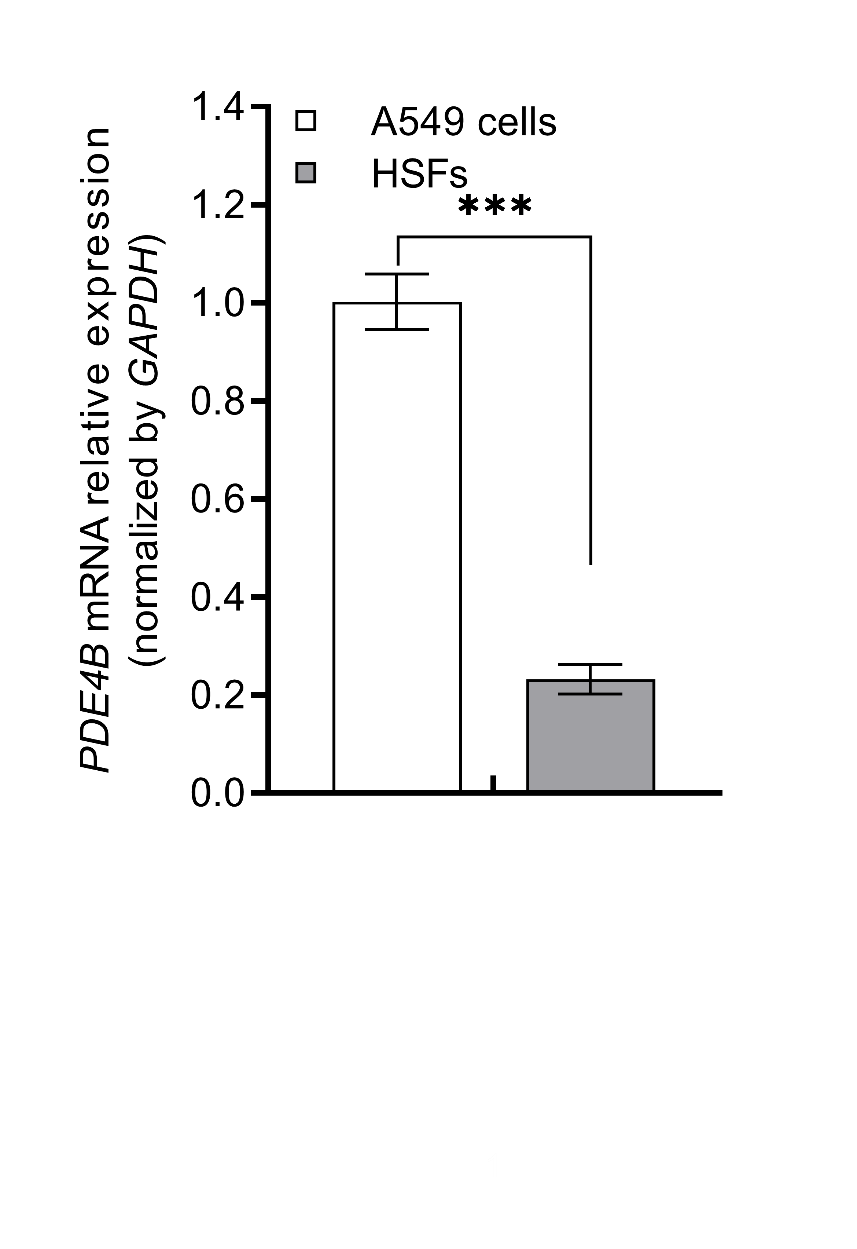


**Figure S3** **|** **Comparison between *PDE4B* mRNA relative expression levels in the HSFs and those in the A549 cell line.** *PDE4B* mRNA expression level in HSFs is only 23% of the value in the value in the normal control A549 cell line. (independent sample *t*-test, ****P* < 0.001).


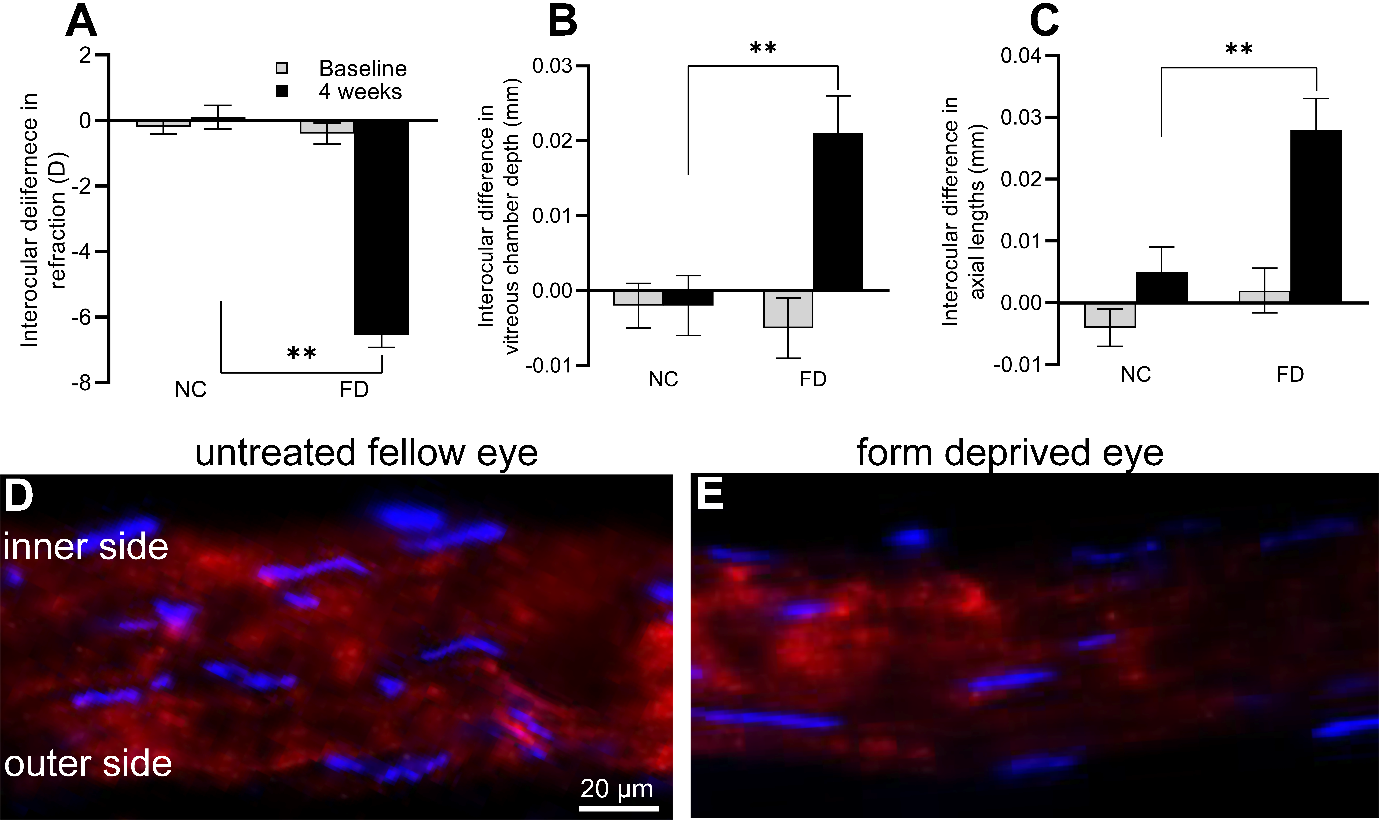


**FIGURE S4** **|** **Refraction, ocular biometrics, and scleral Pde4b expression level in four-weeks form deprivation-induced myopia mice.** Form deprived eyes showed a significantly lower myopia (interocular difference) (**A**), with a shallower vitreous body depth (**B**) and shorter axial length **(C)** compared with untreated fellow eyes (independent samples *t-*test, ***P* <0.01). Comparison of Pde4b immunofluorescent staining patterns in the untreated fellow eye (**D**) and the form deprivation-treated eye (**E**). The Pde4b immunofluorescent staining intensity (red) in the form deprivation-induced eyes (**E**) was significantly lower than that of the untreated fellow eyes (**D**). DAPI staining (blue) positioned nuclei. The images are representative of sections from 3 animals. Scale bar = 20 μm. inner side, the choroidal pigment identifies the inner side of the sclera; outer side, the episclera identifies the outer facing side of the sclera.

**Table S1. qRT-PCR primer sequences for *PDE4B*, *COL1A1*, and *GAPDH* in A549 cells.**

| Gene | Forward primer (5'-3') | Reverse primer (5'-3') | Length (bp) |
| --- | --- | --- | --- |
| *PDE4B* | AACGCTGGAGGAATTAGACTGG | GCTCCCGGTTCAGCATTCT | 110 |
| *COL1A1* | CGAGCGTGGTGTGCAAGGTC | CTGCACCACGTTCACCAGGC | 158 |
| *GAPDH* | GCTCTCTGCTCCTCCTGTTC | GACTCCGACCTTCACCTTCC | 100 |

*GAPDH*, glyceraldehyde phosphate dehydrogenase.

**Table S2. Comparison of estimated allele frequency between pooling control samples in the WMU cohort and Han Chinese Genome Database.**

| CHR | POS | rsID | Minor allele | MAF in pooling | MAF in Han Chinese | *P* adjusted* |
| --- | --- | --- | --- | --- | --- | --- |
| 4 | 73676690 | rs1346132 | G | 5.50% | 2.80% | 0.0009 |
| 1 | 66573381 | rs10889602 | G | 5.50% | 5.50% | 1 |
| 12 | 21047074 | rs4149152 | G | 6.34% | 8.10% | 1 |
| 17 | 76429546 | rs618324 | C | 11.43% | 2.10% | 1.98E-15 |
| 12 | 97563086 | rs2193015 | T | 49.00% | 43.20% | 0.459 |
| 18 | 49625503 | rs9676191 | C | 19.62% | 14.30% | 0.0018 |
| 1 | 53532609 | rs11580093 | C | 5.09% | 3.20% | 0.117 |
| 6 | 16877190 | rs7762018 | A | 44.40% | 29.80% | 1.5822E-13 |
| 11 | 86402877 | rs600242 | G | 14.59% | 16.10% | 0.315 |

CHR, chromosome; POS, physical position of chromosome; rsID, reference SNP number; MAF, minor allele frequency; **P* obtained after Bonferroni correction.
